# Evolution and Mechanism of Spectral Tuning of Blue-Absorbing Visual Pigments in Butterflies

**DOI:** 10.1371/journal.pone.0015015

**Published:** 2010-11-24

**Authors:** Motohiro Wakakuwa, Akihisa Terakita, Mitsumasa Koyanagi, Doekele G. Stavenga, Yoshinori Shichida, Kentaro Arikawa

**Affiliations:** 1 Laboratory of Neuroethology, Sokendai-Hayama, Hayama, Japan; 2 Department of Biophysics, Graduate School of Science, Kyoto University, Kyoto, Japan; 3 Department of Biology and Geosciences, Graduate School of Science, Osaka City University, Osaka, Japan; 4 Department of Neurobiophysics, University of Groningen, Groningen, The Netherlands; Lund University, Sweden

## Abstract

The eyes of flower-visiting butterflies are often spectrally highly complex with multiple opsin genes generated by gene duplication, providing an interesting system for a comparative study of color vision. The Small White butterfly, *Pieris rapae*, has duplicated blue opsins, PrB and PrV, which are expressed in the blue (*λ*_max_ = 453 nm) and violet receptors (*λ*_max_ = 425 nm), respectively. To reveal accurate absorption profiles and the molecular basis of the spectral tuning of these visual pigments, we successfully modified our honeybee opsin expression system based on HEK293s cells, and expressed PrB and PrV, the first lepidopteran opsins ever expressed in cultured cells. We reconstituted the expressed visual pigments *in vitro*, and analysed them spectroscopically. Both reconstituted visual pigments had two photointerconvertible states, rhodopsin and metarhodopsin, with absorption peak wavelengths 450 nm and 485 nm for PrB and 420 nm and 482 nm for PrV. We furthermore introduced site-directed mutations to the opsins and found that two amino acid substitutions, at positions 116 and 177, were crucial for the spectral tuning. This tuning mechanism appears to be specific for invertebrates and is partially shared by other pierid and lycaenid butterfly species.

## Introduction

Visual pigment molecules consist of an opsin, an integral membrane protein with seven transmembrane helices, and a chromophore, most commonly 11-*cis* retinal, which is attached to a lysine in the seventh helix in all opsins so far identified. Upon absorption of a photon, the chromophore isomerizes into the all-*trans* form, which subsequently causes transformation of the whole visual pigment molecule into a metarhodopsin state. This then triggers the intracellular transduction cascade, which eventually produces a receptor potential in the photoreceptor cell. The wavelength range where light effectively isomerizes the chromophore depends on the amino acid residues that interact with the chromophore. Most animals in fact have multiple opsins, which together form the molecular basis of their color vision.

Accumulated evidence indicates that insect opsins can be divided into three clades corresponding to opsins of the ultraviolet (UV)-, blue (B)- and long wavelength (L)-absorbing visual pigment. For example, honeybees employ one opsin from each clade, expressed separately in the UV, B and green (G) receptors in the compound eye [1]. However, this basic pattern is often modified in butterflies, presumably because their life style strongly depends on their color discrimination capacities. Butterfly eyes are in general very rich in terms of the spectral organization, with even more than 6 classes of spectral receptors in some species. This spectral multiplication is partly based on a number of duplication events of the opsins. B opsins duplicated independently in the families Pieridae and Lycaenidae [2], [3], [4], duplication of L opsins was found in Papilionidae [5], [6] and Riodinidae [7], and UV opsins duplicated in the genus *Heliconius* belonging to the Nymphalidae [8].

The opsin is the primary determinant of a photoreceptor's spectral sensitivity. The Small White butterflies, *Pieris rapae*, have four opsins belonging to the ultraviolet (PrUV), blue (PrV, PrB) and long wavelength (PrL)-absorbing visual pigment clades. But interestingly, their eyes have at least 6 classes of receptors, namely the ultraviolet (UV, peak wavelength *λ*_max_ = 360 nm), violet (V, 425 nm), blue (B, 453 nm), green (G, 563 nm), red (R, 620 nm) and deep-red (dR, 640 nm) receptors [9], [10]. The UV, V, B and G receptors express PrUV, PrV, PrB and PrL visual pigments, respectively, and indeed their spectral sensitivity is similar to the visual pigments' absorption spectra. However, the R and dR receptors express the same PrL as that of the G receptors. Their sensitivity peak is strongly shifted to longer wavelengths, which is caused by perirhabdomal, red and deep-red pigments that act as effective red filters [11]. Moreover, the PrV-expressing V receptors of *Pieris* are female-specific. The males also have PrV-expressing photoreceptors, but they are double-peaked blue (dB) receptors due to the filtering effect of a male-specific fluorescing pigment [2]. This proliferation of short-wavelength receptor types emphasizes the biological significance of the duplication of the B opsin as well as the sex-dependent tuning of the spectral sensitivity of the photoreceptors.

To understand the molecular evolution of the biologically significant spectral tuning, direct analysis of purified visual pigment molecules is indispensable. However, notwithstanding numerous attempts for decades, such an analysis has remained unsuccessful for invertebrate visual pigments. Only recently we succeeded to express the UV and B opsins of the Japanese honeybee, *Apis cerana japonica*, in HEK293s cells, which were the first insect opsins ever expressed in cultured cells [12]. Encouraged by this lead, we decided to expand the method to butterflies. We chose the visual pigments of *Pieris rapae*, because their absorption spectra are well-characterized via the photoreceptor spectral sensitivities determined by combined intracellular electrophysiology and optical modeling [2].

As a result, we could express and reconstitute at least the two duplicated B opsins, PrB and PrV. Spectroscopic analyses revealed that the reconstituted PrB and PrV have absorption peak wavelengths at 450 nm and 420 nm, respectively. To identify the amino acid residues responsible for the difference in the *λ*_max_-values, we searched for the candidate positions by aligning the PrB and PrV sequences with a visual pigment of the Japanese Common Squid, *Todarodes pacificus*, whose three dimensional structure was recently determined [13]. We looked for amino acids that are close to the chromophore having different polarity between PrB and PrV, and thus focused on two amino acid residues and tested their contributions by introducing site-directed mutations followed by spectroscopic analyses of the mutant molecules. Moreover, we compared the corresponding amino acids in some other butterfly species to clarify the evolutionary process of butterfly B opsins.

## Materials and Methods

### Animals

We used the Small White butterfly, *Pieris rapae crucivora* Boisduval, from a laboratory culture, which was derived from eggs laid by females captured in Kanagawa, Japan. Hatched larvae were raised under a light regime of 10L14D at 19°C, which induces pupal diapause. The diapausing pupae were kept at 4°C for at least three months, after which they were allowed to emerge at 25°C.

### Expression and purification of expressed visual pigments

The coding region of the cDNAs of the opsins of PrUV, PrB, PrV and PrL visual pigments [2], [11] were amplified from the eyes of *Pieris rapae* by RT-PCR. The amplified cDNAs were tagged by the monoclonal antibody rho-1D4 epitope sequence (ETSQVAPA) [14], [15]. The tagged cDNA was inserted into three different plasmid vectors: pcDNA3.1 containing the human cytomegalovirus (CMV) immediate early promoter (Invitrogen, Carlsbad CA, USA); SRα containing the simian virus 40 (SV-40) early promoter [16]; and EF-1α containing the human elongation factor 1α (Invitrogen, Carlsbad CA, USA). Site-directed mutations were made using a commercial kit, QuikChange (Stratagene). A hundred micrograms of plasmid DNA was used for the transient expression in 10 dishes (*ϕ*10 cm) of cultured human embryonic kidney (HEK) 293s cells. Transfection of plasmid vectors into HEK293s cells was carried out by the calcium-phosphate method [15]. The transfected cells were harvested after 2 days and collected by centrifugation.

The expressed proteins were incubated with excess amount of 11-*cis* 3-hydroxyretinal, the native chromophore of *Pieris* visual pigments [17]. The reconstitution efficiency with 11-*cis* 3-hydroxyretinal appeared to be much lower than when using 11-*cis* retinal, however, and therefore we decided to use 11-*cis* retinal in most of our experiments. The reconstituted visual pigments were extracted with 1% dodecyl β-D-maltoside (DM) in 50 mmol/L HEPES buffer (pH 6.5) containing 140 mmol/L NaCl (buffer A). For purifying expressed proteins from the crude extract, the proteins were bound to agarose beads conjugated with 1D4 antibody, washed with 0.02% DM in buffer A (buffer B) and eluted with buffer B containing the C-terminal peptide of bovine rhodopsin.

### SDS polyacrylamide gel electrophoresis and immunoblotting

The crude extract samples were loaded on 12% SDS polyacrylamide gels and transferred to polyvinylidene difluoride membrane. The membranes were blocked with 1% bovine serum albumin in phosphate-buffered saline containing 0.1% Tween 20 (TPBS) and incubated at room temperature with culture supernatant containing 1D4 antibody overnight. After washing with TPBS, the membranes were incubated with biotinylated secondary antibody. The membranes were then incubated with VECTASTAIN ABC reagent (Vector Laboratories, Burlingame, USA). The membranes were further incubated in peroxidase substrate solution until adequate stain intensity developed.

### Spectrophotometry

Absorbance spectra of the crude and purified samples were recorded at 0°C with a UV2450 spectrophotometer (Shimadzu, Kyoto, Japan). A 1-kW halogen lamp was used for irradiating the samples. Two blue lights (420 and 460 nm) were supplied by the light source with a 460 nm band-pass filter in combination with a Y-43 low-pass filter and with a 420 nm band-pass filter with a L-39 low-pass filter (Toshiba, Tokyo, Japan), respectively. Two green lights (500 and 550 nm) were supplied similarly with a 550 nm band-pass plus a O-53 low-pass filter and a 500 nm band-pass plus a Y-47 low-pass filter, respectively. The full-width half maximum of the band-pass filters was ∼10 nm. Absorbance difference spectra were calculated from the absorbance spectra measured before and after the blue or green irradiations. When necessary, measured absorbance spectra were corrected by subtracting an empirical Rayleigh-scattering baseline.

## Results

### Spectroscopy of expressed PrB and PrV

**Figure 1:**
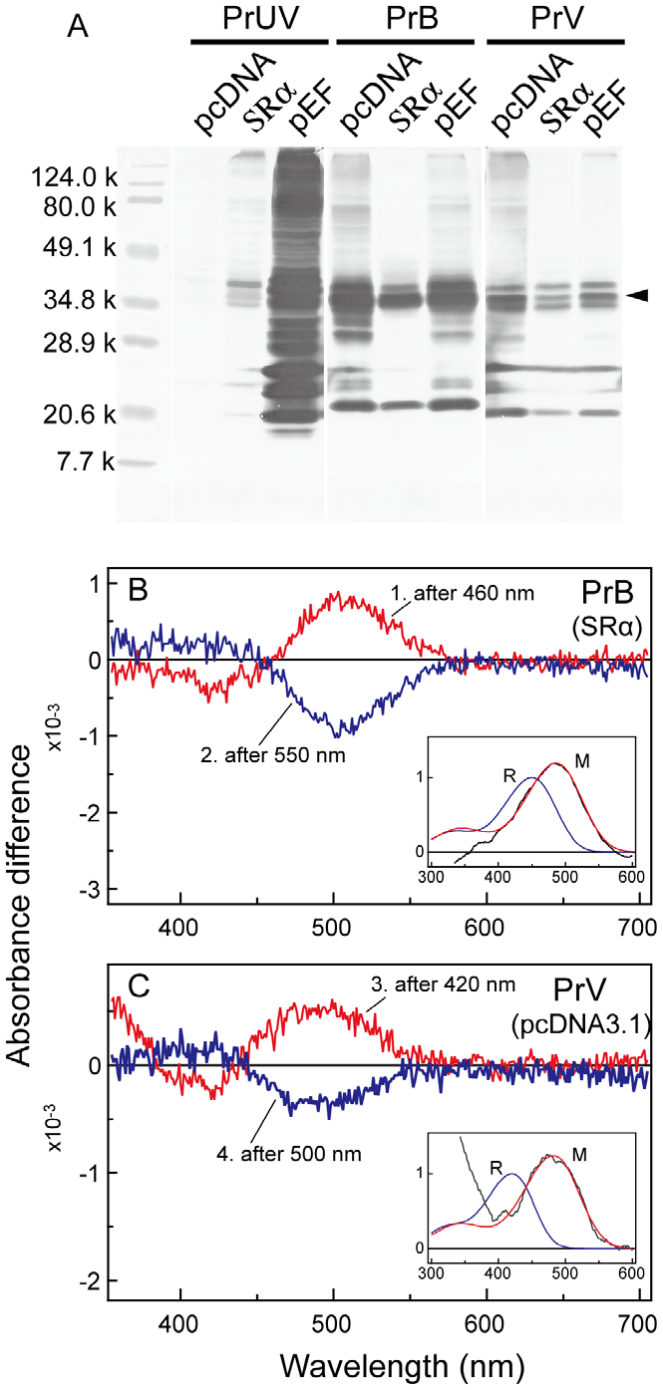
Expression of *Pieris* visual pigments in HEK293s cells. (A) Immunoblot analysis of 1D4 epitope-tagged proteins. The bands of about 38 kD (arrowhead) correspond to the full-length PrUV, PrB and PrV proteins expressed with three vectors, PcDNA, SRα and pEF. (B) Bistability of the crude extracts of PrB. Absorbance spectra were subsequently recorded in the dark, after irradiation with 460 nm and with 550 nm. Curve 1 is the difference before and after 460 nm irradiation, and curve 2 is the difference before and after 550 nm irradiation. The inset shows the average of the two difference spectra (black line), the predicted absorbance spectra of an R450 visual pigment (R) taken from Fig. 2B, and its metarhodopsin peaking at 485 nm derived from the Govardovskii template (M). (C) Bistability of the crude extracts of PrV. Samples were irradiated subsequently with 420 nm and 500 nm. Curves 3 and 4 are the differences of the absorbance spectra before and after 420 nm irradiation and before and after 500 nm irradiation, respectively. The inset shows the average of the two difference spectra (black line), the predicted absorbance spectra of an R420 rhodopsin (R), taken from Fig. 2C, and its metarhodopsin peaking at 482 nm derived from the Govardovskii template (M).

We first confirmed that the HEK293s cell system properly functioned by immunoblot analyses using the anti-rhodopsin 1D4. The 1D4 antibody predominantly labeled bands around 40k (arrowhead in Fig. 1A), which are most likely PrUV, PrB and PrV opsins. We could not detect any bands around 40k for PrL (data not shown) (Fig. 1A). The bands corresponding to smaller and larger molecular weights are probably degraded and aggregated products, respectively.

We incubated the expressed opsins in all vectors with 11-*cis* retinal *in vitro*, extracted the reconstituted visual pigments in the dark, and irradiated the pigments with monochromatic lights to check their photointerconvertibility, which was confirmed only in PrB expressed in SRα and PrV in pcDNA3.1. The extracted PrB (in SRα) and PrV (in pcDNA3.1) were irradiated sequentially with blue light (420 or 460 nm) and green light (500 or 550 nm) between which we recorded absorbance spectra. Using these spectra, we calculated the absorbance difference spectra (Fig. 1B,C), which indicated that PrB and PrV were expressed successfully. The expressed PrB and PrV visual pigments had two photointerconvertible states (Fig. 1B,C).

**Figure 2:**
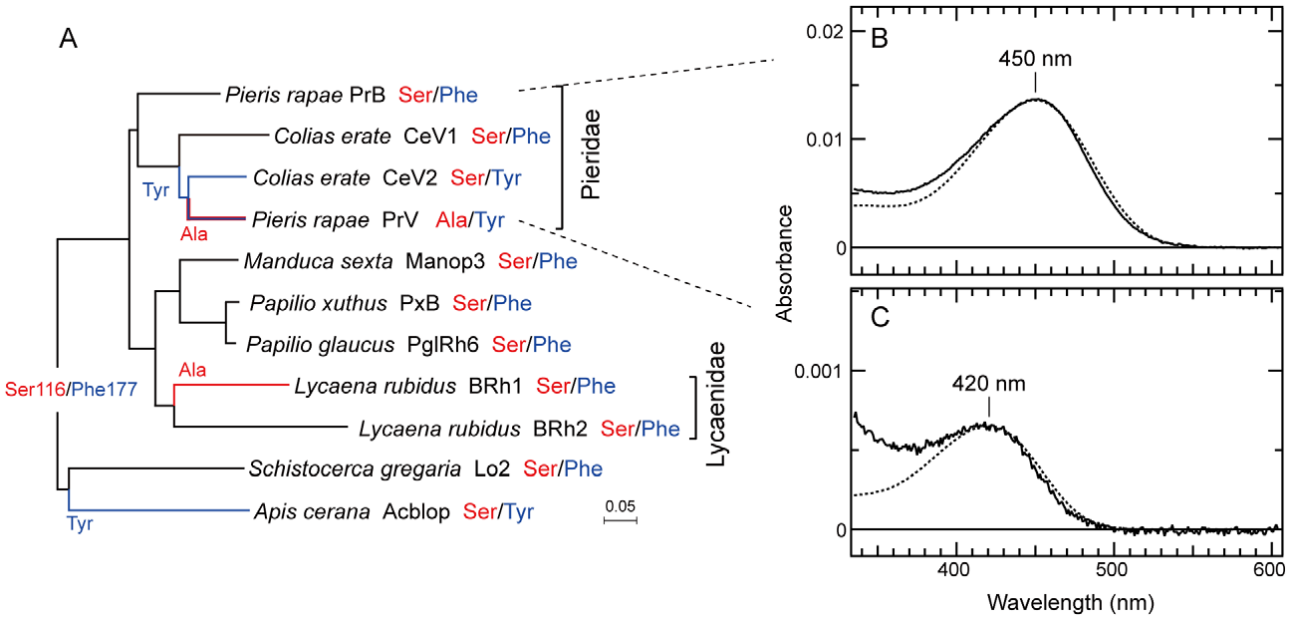
Phylogenic relationship of insect blue opsin and absorption spectra of *Pieris* blue visual pigments. (A) Phylogeny of insect B opsins, constructed with the NJ method. Letters in red and blue indicate the amino acids at 116 and 177, respectively (see Fig. 3A). Red and blue lines indicate the substitution of Ala for Ser at 116 and the substitution of Tyr for Phe at 177, respectively. Only for PrV, both substitutions occurred. (B) Absorbance spectrum of PrB reconstituted with 11-*cis* retinal and purified together with the spectrum predicted for R450 (dotted line). (C) Absorbance spectrum of PrV reconstituted with 11-*cis* retinal and purified together with the predicted for R420 (dotted line).

After confirming photoconversions in the crude extracts, we purified the expressed visual pigments and measured the absorbance spectra of reconstituted and purified PrB and PrV (Fig. 2). Figure 2 also shows the phylogenetic relationship of the opsins with other insect B opsins (see Arikawa et al. 2005). The absorbance spectra of the purified PrB and PrV were fitted by eye with a visual pigment template [18] yielding peak wavelengths 450 nm and 420 nm, respectively. We note here that the visual pigments were reconstituted using 11-*cis* retinal (A1 retinal), but in fact the native chromophore of butterfly visual pigments is 11-*cis* 3-hydroxyretinal (A3 retinal) [17]. To uncover possible spectral effects due to the different chromophores, we repeated the reconstitution using A3 retinal. The resulting absorbance spectra appeared to be very similar to those measured with A1 retinal, but the reconstitution efficiency was much lower with A3 retinal due to unknown reasons, and therefore we could not accurately determine the *λ*_max_ values.

Using the absorbance spectra of PrB and PrV determined in purified samples, we calculated the absorbance characteristics of their metarhodopsin states based on the difference absorbance spectra (Fig. 1). We took the spectrum of R450 predicted by the Govardovskii template [18], added the normalized average of two difference spectra for PrB (Fig. 1B) multiplied by factors in the range 1.5±0.5, and then chose the best-fitting curve using again the Govardovskii template; the template describes metarhodopsin spectra reasonably well [19]. We thus found that the metarhodopsin of PrB has a peak wavelength around 485 nm (Fig. 1B, inset). Applying the same procedure with a rhodopsin R420 for PrV yielded a metarhodopsin with peak wavelength around 482 nm (Fig. 1C).

### Effects of site-directed mutagenesis

**Figure 3:**
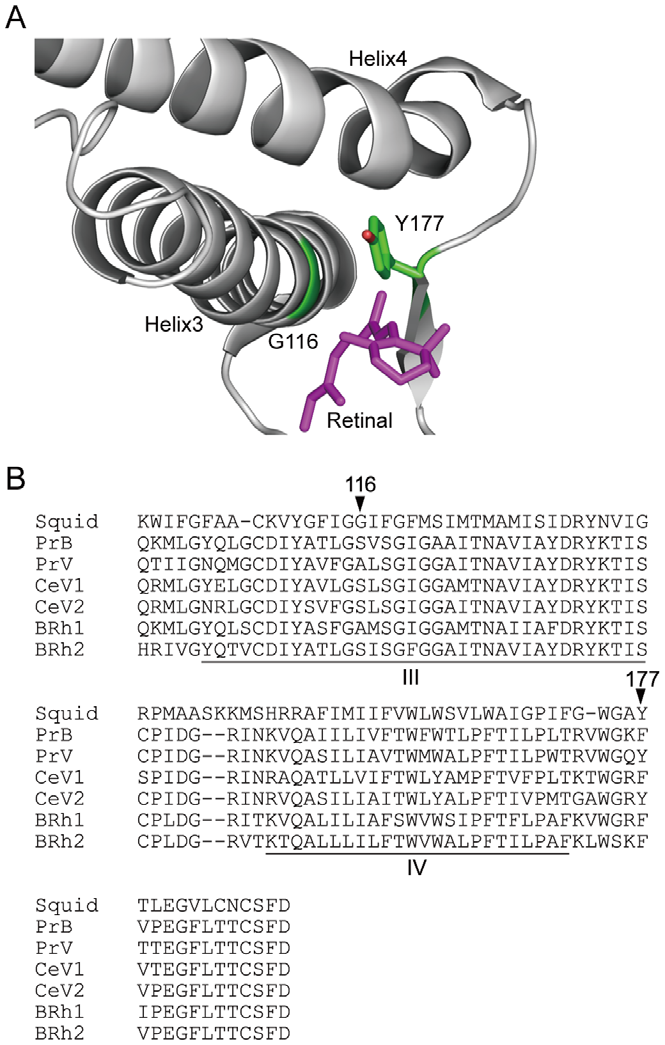
Amino acids responsible for spectral tuning in butterfly B opsins. (A) Partial 3D structure of the rhodopsin of the Common Squid, *Todarodes pacificus*, and the positions of Gly116 and Tyr177 (14). (B) Alignment of partial amino acid sequences of butterfly B opsins with the squid opsin. Arrowheads indicate the amino acid residues replaced in the mutation experiments. See Fig. 2 for opsin names. III and IV, third and fourth transmembrane regions.

To analyze the mechanism underlying the observed difference in *λ*_max_ of the wild type PrB and PrV, we analyzed site-directed mutants of these visual pigments. First we aligned the amino acid sequences of PrB and PrV with that of the rhodopsin of the Japanese Common Squid, *Todarodes pacificus*, because the 3D structure of this rhodopsin is known [13] (Fig. 3). We then searched amino acid residues located within 5 Å from any carbon of the chromophore, and found 24 residues in total. Among the 24 residues we identified two residues having different polarities between PrB and PrV, which were Ser116 (PrB) vs Ala116 (PrV) and Phe177 (PrB) vs Tyr177 (PrV) (Fig. 3B, arrowheads, numbering according to the squid rhodopsin). Next we genetically manipulated the opsins; for PrB, Ser116 and/or Phe177 were replaced by Ala and/or Tyr, and for PrV, Ala116 and/or Tyr177 were replaced by Ser and/or Phe. We expressed these mutant visual pigments using SRα for the PrB mutants and pcDNA3.1 for the PrV mutants. We then analyzed them spectroscopically in the native, dark state.

**Figure 4:**
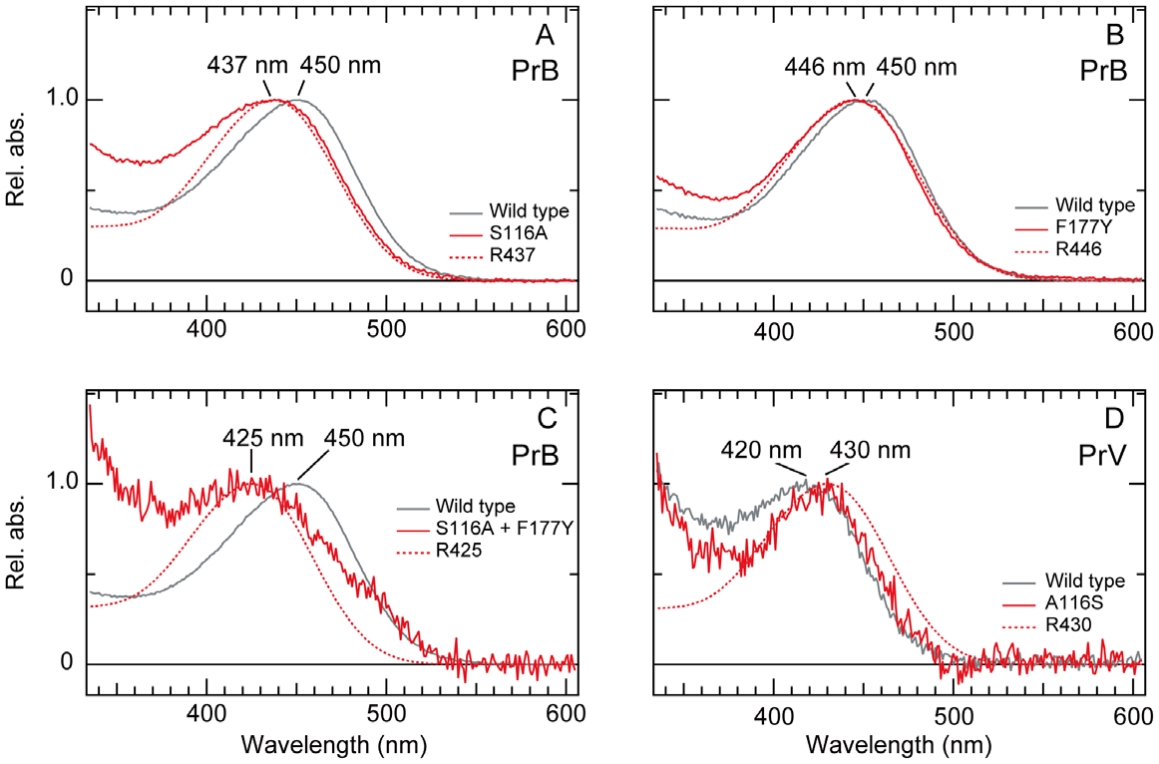
Absorbance spectra of purified PrB and PrV and their mutants with spectra predicted by the Govardovskii template (dotted lines). (A) Absorbance spectra of the wild type PrB (black line) and of the mutant whose Ser at position 116 was substituted with Ala (S116A, red line). (B) Absorbance spectra of wild type PrB (black) and the Phe177Tyr mutant (red). (C) Absorbance spectra of wild type PrB (black) and the double mutant Ser116Ala and Phe177Tyr (red). (D) Absorbance spectra of the wild type PrV (black) and its mutant Ala116Ser (red).

The normalized absorbance spectra of the mutant pigments (red lines) with predicted spectra based on the Govardovskii template (dotted lines) are shown in Fig. 4. In both PrB mutants, the *λ*_max_ shifted to shorter wavelengths: substituting Ser116 to Ala resulted in a 13 nm hypsochromic shift, from 450 nm to 437 nm (Fig. 4A, red line), and substituting Phe177 to Tyr resulted in a 4 nm shift, to 446 nm (Fig. 4B, red line). In these single mutants of PrB, the absorbance spectra matched well with the predicted spectra. However, the expression level of the double mutant of PrB and all mutants of PrV was rather low, indicated by the noise of the recorded spectra (Fig. 4C,D). In the double mutant of PrB, Ser116Ala plus Phe177Tyr, *λ*_max_ shifted 25 nm hypsochromically to 425 nm (Fig. 4C). This value approximates the *λ*_max_ = 420 nm of the expressed wild type PrV (Fig. 4D, black line). For PrV, we could only record a spectrum for the mutant Ala116Ser. This mutant exhibited a 10 nm bathochromic shift of *λ*_max_ to 430 nm (Fig. 4D, red line), a shift close to that of the reverse case, i.e., the Ser116Ala substitution in the PrB mutant (Fig. 4A). The noisy spectra did not match with the predicted spectra, which will presumably be improved with increasing expression level. Taken together, the two amino acid residues at positions 116 and 177 are most likely responsible for the spectral tuning of the blue absorbing visual pigments of *Pieris rapae*.

## Discussion

### Absorbance spectra of the native PrB and PrV

After a long struggle to express insect visual pigments in cultured cells in the past decades, Terakita et al (2008) were finally successful in expressing the ultraviolet- and blue-absorbing visual pigments of the Japanese honeybee, *Apis cerana japonica*. The present account is the second report of this line of study. Based on the present spectroscopic analyses of reconstituted PrB and PrV, we have concluded that PrB is a visual pigment with peak absorbance at 450 nm (R450) and that PrV peaks at 420 nm (R420; Fig. 2B,C). The absorbance peak wavelengths of their metarhodopsin states are 485 nm for PrB and 482 nm for PrV (Fig. 1B,C).

**Figure 5:**
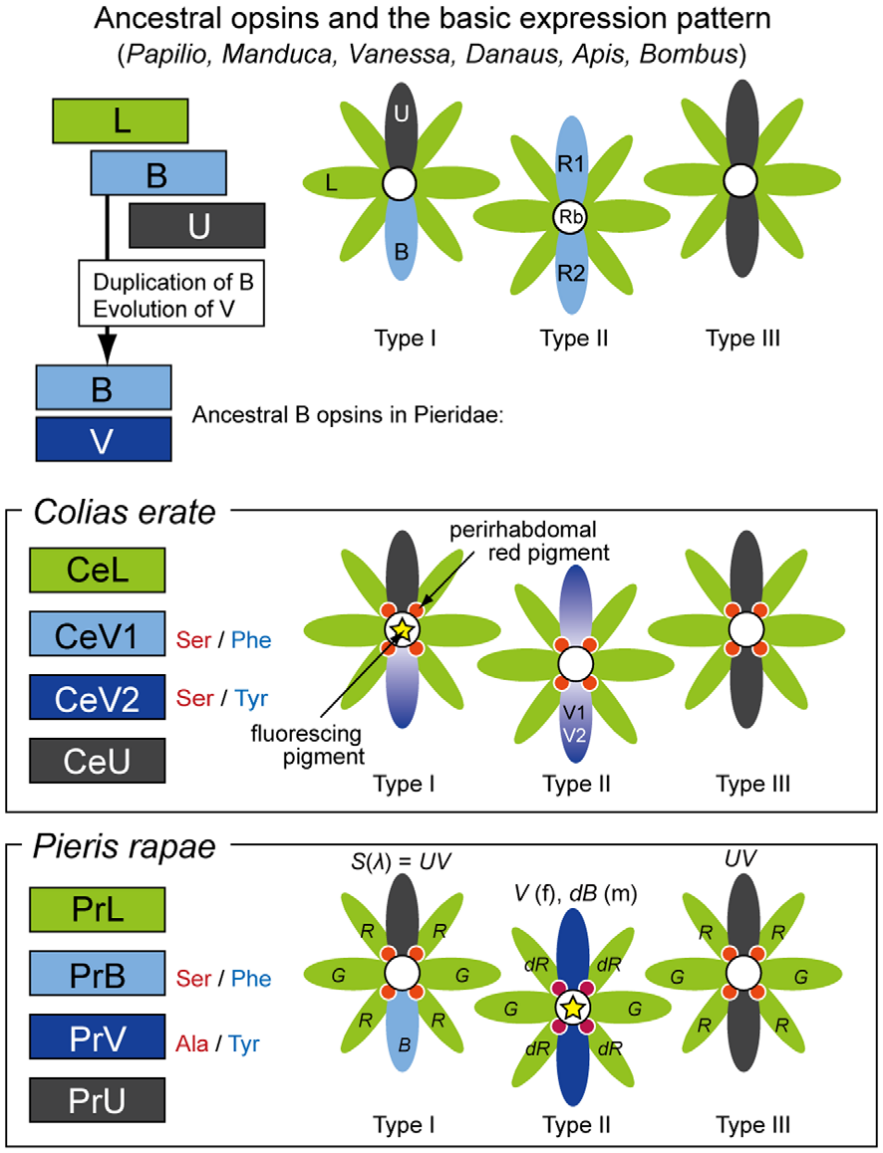
Evolution of B opsins in Pieridae. The top panel shows the ancestral set of opsins and their expression pattern in three ommatidial types. The ancestral pattern with three opsins, U, B and L, in three fixed combinations is retained in a number of insect species; e.g. the butterflies *Papilio xuthus* [27], *Vanessa cardui* [28], *Danaus plexippus* [29], the moth *Manduca sexta* [30], and the bees *Apis mellifera* [1] and *Bombus impatiens* [31]. In the lineage of Pieridae, the B opsin duplicated, forming the ancestral B and V opsins. The middle and bottom panels show the opsins and ommatidia of *Colias erate* [3] and *Pieris rapae* [2], [11], showing subfunctionalization of photoreceptors R1 and R2. The *Colias* eyes have two V opsins, CeV1 and CeV2, coexpressed in a subset of R1/R2: the B opsin was lost in *Colias* (see Fig. 2A). In *Pieris*, the B opsin in one of the ancestral ommatidial types is replaced by the V opsin, PrV. The photoreceptor spectral sensitivities were further modified by perirhabdomal red pigments (indicated by red dots), and in males by a fluorescing pigment in the rhabdom (yellow star in type II). A fluorescing pigment is also present in *Colias* in ommatidial type I. Capital letters and colors indicate names and types of the opsins. Italic letters indicate spectral sensitivities (*S*(*λ*): *UV*, ultraviolet; *B*, blue; *dB*, double-peaked blue; *G*, green; *R*, red; *dR*, dark red). Red and blue letters indicate amino acid at positions 116 and 177, respectively (see Fig. 2A).

We identified the photoreceptors expressing these visual pigments [2], [11] and also electrophysiologically measured their spectral sensitivities [9], [10]. The sensitivity profile of the PrB-expressing photoreceptors well matched the calculated absorbance spectrum of a rhodopsin R453 [2]. The spectral sensitivity of the PrV-expressing photoreceptors of females peaks at 425 nm and corresponds to the absorbance spectrum of a rhodopsin R425. However, the PrV-expressing photoreceptors of males are double-peaked blue (dB) receptors, peaking at 460 nm [2], [9]. This is due to a male-specific fluorescing pigment in the rhabdom, which acts as a short-wavelength absorbing, long-pass filter for the colocalized photoreceptors, and thus modifies the PrV-expressing photoreceptors in the fluorescing ommatidia into dB receptors (Fig. 5).

The peak absorbance wavelengths of PrB and PrV were a few nm shorter: 453 vs 450 nm for PrB and 420 vs 425 nm (Fig 2BC). Presumably these spectral differences are due to the lens-waveguide optics of the butterfly compound eye, or may be due to the A1 retinal used to reconstitute the visual pigments instead of their native A3 retinal chromophore.

Establishment of an expression system in cultured cells has been a major hurdle in the study of spectral tuning of butterfly photoreceptors, which has become a central topic of the evolution of color vision. Although the HEK293s system has finally proved to be successful in expressing the short-wavelength visual pigments of two insect species, the bee *Apis cerena* and the butterfly *Pieris rapae*, it still requires further improvement. For instance, we have tried to express PrL, the opsin of the long-wavelength absorbing visual pigment of *Pieris rapae*, in the same system, but, as was also experienced in *Apis cerana*, we could not detect any sign of a functional visual pigment. This indicates that the L-type opsins expressed in cultured cells are susceptible to denaturation during post-translational modification. Co-expression of an adequate molecular chaperone may perhaps solve this technical problem.

### Molecular basis of the spectral tuning

We carried out spectroscopy of the reconstituted PrB, PrV and their site-directed mutants. Analyses of mutated insect visual pigments have only been reported for the *Drosophila* UV and G opsin mutants, which were ectopically expressed in *Drosophila* R1-6 photoreceptors [20], [21]. To the best of our knowledge, the present account is the first report based on *in vitro* reconstitution and analyses of mutant visual pigments of invertebrates.

PrB and PrV, sharing 78% of amino acids, are produced by a gene duplication event. To find the amino acid residues responsible for the spectral tuning, we surveyed the amino acid sequence of PrB and PrV in comparison with that of the squid rhodopsin (Fig. 3A). Among several candidates localized within a range of 5 Å from any carbon in the chromophore, we selected two amino acids whose properties were different between PrB and PrV. These residues were Ser116 and Phe177 in PrB and Ala116 and Tyr177 in PrV, corresponding to Gly116 and Tyr177 of the squid rhodopsin. In squid, the distance between the α carbon of Gly116 and the C19 methyl carbon of 11-*cis* retinal is 3.47 Å, and the distance of the hydroxyl group of Tyr177 and the C19 methyl carbon of 11-*cis* retinal is 4.04 Å.

Here we found that the substitution of Ser to Ala in PrB at position 116 caused a 13 nm hypsochromic peak wavelength shift (Fig. 4A), whereas the substitution of Ala to Ser in PrV caused a 10 nm bathochromic shift (Fig. 4D). This indicates that the hydroxyl group of Ser at 116 interacts with the chromophore. A similar phenomenon has been observed in bovine rhodopsin, which has Thr at 118 (equivalent to squid 116). The substitutions Thr118Ala and Thr118Val, both removing the hydroxyl group, resulted in an 18 nm and 15 nm hypsochromic shift, respectively [22]. Although these substitutions were induced experimentally, they appear not to have happened in nature: all wild type visual and non-visual opsins of vertebrates have Thr or Ser, both with a hydroxyl group, at the corresponding positions. This suggests that the spectral shift due to removing a hydroxyl group at 116 specifically occurs in invertebrate visual pigments.

The *λ*_max_ of the Phe177Tyr mutant of PrB exhibited a 4 nm hypsochromic shift (Fig. 4B). Although the reverse case, the Tyr177Phe mutant of PrV, could not be successfully expressed, the effect of a hydroxyl group at 177 is probably opposite to that of a hydroxyl group at 116, i.e. it will cause a bathochromic shift. So far, there was no concrete information about the role of the amino acid at 177 for spectral tuning in any visual pigment. Indeed, in bovine rhodopsin the amino acid at 178 (equivalent to squid 177) is located rather far from the chromophore (6.26 Å), but in the squid it is closer (4.04 Å), suggesting that the role of the amino acid residue at 177 is also specific to invertebrates.

### Evolution of blue visual pigments in butterflies

Figure 2A shows a phylogenetic relationship of B visual pigments of lepidopteran species. The most common pattern is Ser and Phe at positions 116 and 177, indicating that this is the ancestral combination in insect B opsins. Duplication of B opsin appears to have happened in the families Pieridae and Lycaenidae.

The evolution of opsins and the spectral organization of insect visual systems are summarized in Fig. 5 with a particular emphasis on the B opsins of Pieridae. A number of species, including butterflies, moths and bees, have three opsins of the UV, B and L type. The opsins are expressed in the nine photoreceptors of each ommatidium in three different patterns, making the eye a mesh of three spectrally heterogeneous ommatidia (Fig. 5, top panel). The heterogenous organization of the eye with three ommatidial types is shared by many species so far studied, and therefore appears to be ancestral. Below the ancestral pattern, Fig. 5 shows actual examples of eye organization of two pierid species, *Colias erate* and *Pieris rapae*.

Starting from the ancestral pattern, the ancestral B opsin duplicated in the lineage of Pieridae. The duplicated B opsin accumulated amino acid substitutions forming the ancestral V opsin, which still retained Ser116 and Phe177. The phylogeny of opsins (Fig. 2A) indicates that in Coliadinae the ancestral B was somehow removed and that the ancestral V duplicated again. In *Colias erate*, CeV1 and CeV2 evolved from the duplicated V opsins. In the process of CeV2 evolution, the substitution Phe177Tyr took place, which most likely shifted the absorption peak wavelength several nanometers hypsochromically (see Fig. 4B). The V opsin of *Pieris*, PrV, evolved from the common ancestor of CeV2 (see Fig. 2A), by substituting Ser116Ala, which caused an additional 19 nm hypsochromic shift (see Fig. 4C).

Two B type opsins, BRh1 and BRh2, have been identified in a lycaenid species, *Lycaena rubidus* [23]. Their absorption maxima were estimated as 437 nm (BRh1) and 500 nm (BRh2) by microspectrophotometry of the intact eye. Judging from the substitution at 116, BRh1 corresponds to PrV (Ala), whereas BRh2 corresponds to PrB (Ser), matching well with the present results. Because the B opsin duplication events in Pieridae and Lycaenidae are thought to have occurred independently [24], the contribution of the amino acid at 116 to spectral tuning in both families is probably a consequence of parallel evolution. We note here that the difference in the *λ*_max_ of the two visual pigments of *Lycaena* (63 nm) is much larger than in *Pieris* (33 nm), implying that *Lycaena* employs an additional mechanism that functions in an additive manner.

The evolution of the opsins in the butterfly eyes affected of course their spectral organization. In *Colias*, the mRNAs of CeV1 and CeV2 opsins are always coexpressed in a subset of R1 and R2 photoreceptors [3], suggesting that these receptors have a broad-band sensitivity. Indeed, we have encountered broad-band blue receptors in our electrophysiological study on *Colias* eyes [25]. In *Pieris rapae*, the evolution of PrV resulted in subfunctionalization of short wavelength receptors, R1 and R2 (Fig. 5). The type II ommatidia of *Pieris* express PrV both in R1 and R2, which in the ancestral form expressed B opsin. Interestingly, the PrV-expressing photoreceptors are sexually dimorphic due to the male-specific fluorescing pigment [2]. Acquisition of PrV and the sexual dimorphism of spectral sensitivity may be crucial in mate recognition that is driven by the female-specific UV wing reflection of *Pieris rapae crucivora* [26].
